# Effect of palliative care training on perceived self-efficacy of the nurses

**DOI:** 10.1186/s12904-020-00567-4

**Published:** 2020-05-04

**Authors:** Fatemeh Dehghani, Maasoumeh Barkhordari-Sharifabad, Maryam Sedaghati-kasbakhi, Hossein Fallahzadeh

**Affiliations:** 1 Department of Nursing, School of Medical Sciences, Yazd Branch, Islamic Azad University, Shohadaye Gomnam Blvd., Safaiyeh, Yazd, 8916871967 Iran; 2 Department of Nursing, School of Medical Sciences, Tonekabon Branch, Islamic Azad University, Tonekabon, Iran; 3 Department of Biostatistics and Epidemiology, Faculty of Health, Shahid Sadoughi University of Medical Sciences and Health Services, Yazd, Iran

**Keywords:** Palliative care, Self-efficacy, Psychological support, Symptom management, Nurses

## Abstract

**Background:**

Nurses are involved in providing end-of-life care for end stage individuals and their self-efficacy is one of the key factors bearing on such care. The purpose of this study was to determine the effect of palliative care on perceived self-efficacy of the nurses.

**Methods:**

This is a quasi-experimental study with pretest-posttest design. Sampling was randomized and included 40 individuals. The intervention consisted of palliative care training for four sessions, each lasting 45 min. Data were collected using demographic and perceived self-efficacy questionnaires completed before and after the intervention. Data were then analyzed by SPSS 16 software using descriptive and inferential statistics.

**Results:**

The mean age of the participants was 38.6 and their work experience was 14.25 years. The majority of the participants were female (85%) and had a bachelor level of education (92.5%). The findings showed that “perceived self-efficacy”, “psychosocial support” and “symptom management” improved significantly after intervention (*p* < 0.05).

**Conclusion:**

Based on the results, palliative care education has the potential to increase nurses’ perceived self-efficacy. Since all members of the health care team Including nurses play an important role in providing palliative care, nursing managers can take an effective step to maximize the capacity of nurses by planning and supporting training in this regard.

## Background

Care is at the core of the nursing profession and promotes the uniqueness of the profession [1]. Nurses play a key role in caring for end-of-life patients and their families as part of the care team [2]. One type of nursing care is palliative care. According to the World Health Organization, palliative care is an approach that addresses the quality of life of the patients and their families in facing problems related to life-limiting diseases through preventing and improving the patient’s suffering and by identifying early identification and management of pain and other physical, psychological problems which promote the patients’ spiritual and social well-being [3].

About 40 million people worldwide are reported to need palliative care every year, but only 14% of these clients receive intervention of this type [4]. Globally, no communicable diseases cause 70% of deaths and engender 93% of adult palliative care needs. Approximately, 37.4% of all deaths require palliative care and 80% of global palliative care needs are pertinent to middle-income countries [5]. Nurses play an important role in care goals as the individuals on the palliative care team, so their productivity is of critical importance [6].

Studies have shown that although palliative care in the later stages of life is what the patients and their families highly deserve, most nurses are not adequately prepared to provide this type of care, so its provision is becoming more difficult for the nurses [1]. Much research has also been performed on the inadequacy of palliative care education in medical departments revealing that nurses are yet to be well versed in palliative care and thus need more appropriate training for this concept and specific care [7].

The result of a systematic review study revealed limited studies around interventions as to improving palliative care for older people living in nursing homes, all of which accomplished in the United States hence requiring high quality research into palliative care interventions especially outside the United States [8]. The results of a meta-analysis study indicated the positive and effective impact of interprofessional education on knowledge, attitude and skills of students in different health care fields. The researchers suggest further clinical trials to identify the impact of an interprofessional education on students’ clinical ability [9]. Another systematic review found interprofessional education bearing positive results, given the small number of studies and the heterogeneity of interventions and outcome measures, generalization s appear not to be reasonable [10]. On the other hand, ongoing advances in medical technology and patient care have made nursing a complex and promising profession. Effective, advanced and reliable nursing requires skills such as problem solving and the ability to make clinical decisions [11].

What is certain is that knowledge without action is insufficient, and what mediates between knowledge and practice is self-efficacy, and some believe that there is a close relationship between self-efficacy and individual performance in performing the assigned tasks [12]. The concept of self-efficacy is the ability to perform a given task and to successfully perform a certain behavior [13], that has the greatest impact on the nursing performance. High self-efficacy improves quality of care and ultimately improves individual and organizational performance [14–16]. Self-efficacy is also an important predictor of nurses’ behaviors. Research has shown that nurses with higher self-efficacy perceptions provide better quality care than nurses with lower capacity. On the other hand, these nurses are more committed to their work and more resistant to problems [17–19]. Moreover, self-efficacy is an important prerequisite for behavior change [20].

In Iran, palliative care is a new approach [21], and the current situation of palliative care services for Iranian patients is quite different. In this setting, it is the family members who are responsible for caring for patients with incurable diseases, especially those in the end stages. Although all hospitals accept these patients and provide them with their services, but dedicated services are very limited for them. These patients can only be admitted to hospitals for receiving routine services, i.e., those provided to any other patients [22]. In other words, patients in need of special palliative care as well as routine nursing care are admitted to the same ward (usually ICU or oncology ward). Therefore, nurses working in such wards should focus on caring for a considerable number of patients in critical situations so that they are required to have a slew of information; consequently, they fail to focus on attending the people who need special palliative care [23]. Despite this, palliative care education has not been included as a special education course in the undergraduate nursing curriculum [24]. Lack of several critical factors including a palliative care curriculum, appropriate educational content, familiarity with the basic principles of palliative care in health care personnel, and public awareness about these services are considered as a defect in the Iranian educational system [21].

Hence, due to the need of the patients as well as the society for such specialized services, designing such caring programs for nurses involved in the matter is as important as their professional training and should thus be included in nursing education programs to come. In this study, we aimed to study the effect of palliative care training on perceived self-efficacy of the nurses.

## Methods

### Design and samples

This quasi-experimental query was performed on 40 nurses working in Imam Jafar Sadegh Hospital in Meybod, Yazd, Iran. Inclusion criteria were: willingness to participate in research, 1 year of work experience, and ability to attend all the training sessions. Sixty-eight nurses met these criteria, 40 of whom were randomly selected. Regarding the significance level of 5% and test power of 80% and according to a similar work conducted by Joy [25] with s = 6 and significant difference of at least 4 points in the mean score of self-efficacy, 36 cases proved to be needed. So by considering 10% loss, 40 patients were evaluated.

$$ n=\frac{{\left({z}_{\alpha /2}+{z}_{\beta}\right)}^22{S}^2}{{\left({\mu}_1-{\mu}_2\right)}^2} $$

There were no drop-outs in the present study. Participants were selected from CCU, ICU and medical units. As noted earlier, there are limited centers for palliative care throughout Iran and patients in need of such services including cancerous, incurable as well as chronic cases are often admitted to general hospital units. Moreover, in a score of cities, hospitals are small and patients with various problems are generally admitted to the medical units.

In order to achieve the goals of the study, once the informed consent was obtained from the participants, the questionnaires were provided to the nurses before and after the intervention and completed by the nurses participating in the study with an approximate duration of 15 min.

### Intervention

On the basis of need, assessment and goals, four 60–90 min educational workshop were developed by the researcher (the first author) to be completed during 2 days. This author holds M.Sc. in medical-surgical nursing and has passed a palliative care course in theory and internship and bears around 10 years of experience working in diverse parts of the said hospital. Meetings were held in the conference room of the hospital. Session content was based on the training program presented by the Ministry of Health and Medical Education of Iran about “palliative care and the role of the nurse in it”.

In the first session, stated topics comprised basic concepts of palliative care such as principles, goals, and components of supportive, spiritual, and ethical of palliative care, the role of hope, courage and spirituality in enhancing quality of life.

The second session subsumed pain management, identifying its sources and causes and describing pain, determining pain assessment methods, organizing methods of treatment and evaluation of pain, including drug and non-drug treatment.

The third session focused on role of the nurse in ccontrolling the common physical and mental symptoms of the end stages of life, such as nausea, vomiting, insomnia, anxiety, etc.

The fourth session touched upon communication skills needed to manage clients and families in need of palliative care, communication methods with other healthcare providers, how to communicate principles and practices of palliative care.

### Data collection tools

The data collection tool was a two-part questionnaire.

1. Demographic characteristics included 4 questions including age, gender, education level, work experience.
2. Perceived Palliative Care Self-efficacy Questionnaire: This scale was first designed and validated by Phillips et al. [26]. In this study, the scale was translated by the researcher and used, for the first time, in Iran. It focuses on perceived ability to manage common dimensions of end-of-life care. The scale consists of 12 questions having two theoretically distinct dimensions regarding perceived abilities to provide care. The first dimension is “psychosocial support” and the second is “symptom management”. This scale is based on a four-point Likert scale. The highest score is 48 and the lowest is 12. Higher scores were considered as higher self-efficacy. In this study, content and face validity were evaluated using the judgment of experts and quantitative validity indices containing CVR and CVI. The mean content validity index was 1 and the content validity index was appropriate for the whole scale in the present study. After determining the content validity index and content validity ratio to evaluate the reliability of the tool based on the internal consistency method of the items and calculating the alpha coefficient, 30 random samples from the statistical population were studied. It should be noted that these 30 individuals were not included in the sample. Cronbach alpha coefficient was calculated as 0.848 for the first six items (first dimension), 0.78 for the next six items (second dimension) and 0.704 for the overall Cronbach alpha.

### Data analysis

All questionnaires were coded and provided to the participants before data collection and data extraction. Data were then analyzed by SPSS software version 16 using descriptive and inferential statistics (independent t and paired t). The significance level was set at 0.05. Normal distribution of the data was confirmed by Kolmogorov-Smirnov (KS) test before performing the tests (*p* > 0.05).

## Results

The mean age and work experience of the participants reached 38.6 and 14.25 years, respectively. The majority of the participants were female (85%) and had a bachelor level of education (92.5%) (Table 1).

**Table 1:** Demographic characteristics of the sample

Variables	Mean (SD)	N (%)
Age	38.6 (6.05)	
Work experience	14.25 (6.15)	
Gender		
Male		6 (15)
Female		34 (85)
Level of education		
B.Sc.		37 (92.5)
M.Sc.		3 (7.5)

The mean score on perceived self-efficacy after training (39.4 ± 6.9) was significantly higher in the intervention group than before (27.7 ± 7.9) and the difference was statistically significant (*P* = 0.00).

The mean score below the “Symptom Management Dimension” scale after training (20.5 ± 2.4) was higher than that of before training (13.9 4 4.8) and the difference was statistically significant. (*P* = 0.01).

The mean score on the subscale of “psychosocial support” after training (19.3 ± 3.3) was higher than that of before training (13.8 ± 4.03); this was also statistically significant (*p* = 0.001) (Table 2).

**Table 2:** Comparison of perceived self-efficacy before and after the intervention

Variable	Mean (SD)	df	***P*** value
Self-efficacy		39	0.00
Pretest	27.7 (7.98)		
Posttest	39.6 (4.9)		
Symptom Management		39	0.01
Pretest	13.9 (4.8)		
Posttest	20.5 (2.4)		
psychosocial support		39	0.00
Pretest	13.8 (4.03)		
Posttest	19.1 (3.3)		

The results indicated no significant relationship between perceived self-efficacy of the nurses with their work experience, age, education level and gender (*P* < 0.05).

## Discussion

The purpose of this study was to investigate the effect of palliative care training on self-efficacy of nurses. Results demonstrated that palliative care training improves nurses’ perceived self-efficacy score. These results were in line with those of Joy’s [25], which examined the impact of palliative care intervention on increasing the knowledge and self-efficacy of nurses working in long-term care units. In that study, self-efficacy score was 39.9 ± 6.96 in pre-test whereas in post-test it leveled at 44.1 ± 4.08 and the difference was significant. The results of Wen’s et al. [27] study were also in line with those of these results. The findings of Kassa’s study [28] indicated knowledge and functional aspects of palliative care knowledge being poor in Ethiopian nurses but their attitude toward palliative care being appropriate. However, their study was descriptive cross-sectional and did not test an intervention. Since self-efficacy is the most important determinant of behavior change, it can influence one’s choice in the behavioral process and also make the individual spend more effort on the action, so paying attention to it seems to be highly essential.

Not surprisingly, nurses trained in palliative care bear higher self-efficacy [29]. Knowledge is considered a stable and fundamental element for nursing performance. Enjoying knowledge increases the ability to perform, and thus bears an indirect impact on self-efficacy which leads to optimal performance [30]. Self-efficacy predicts nurses’ individual and professional behaviors and affects an individual’s effort and commitment to achieve goal behavior [26]. High self-efficacy improves quality of care and ultimately improves individual and organizational performance.

Unfortunately, palliative care education has not yet been widely implemented in nursing education programs. These programs often focus on acute patient care and fail to regard patients in the end stage of life. As a result, nurses who are unaware of palliative care may not be prepared to cope with patients who are passing out [29, 31, 32].

Another finding of the study was that palliative care training improves the “symptoms management” of perceived self-efficacy. The results of Mokhtar and Abdelmalik’s study are in agreement with those of the present study. In a quasi-experimental study, they examined the effect of a training program on nurses’ awareness of pain management in cancer patients. The results revealed the mean score of the nurses’ knowledge in pre-test 47.99 ± 5.85, after training i.e., in post-test increasing to 81.53 ± 5.75 and after 3 months 94.47 ± 5.67 so that there was a significant difference between pre-test and post-test results. In their study, the employed curriculum had a significant effect on improving nurses’ knowledge of the concepts, assessment and management of cancer pain [33]. In a study carried out by Saadati et al., Nurses’ awareness of pain physiology, pain symptoms and symptoms before training was 32%, which increased to 87.7% after training. Nurses’ perceptions of pain assessment methods and its severity increased from 54.3% before intervention to 85.3% after training. Educational intervention has a role in increasing the knowledge and improvement of nurses’ performance and attitude, which is consonant with the results of our study [34]. The findings of the study by Di Giulio et al., which examined the impact of nursing home staff training on end-of-life care for dementia patients, accords with the results of the present study identifying that short-term educational intervention modifies quality-related interventions for end-of-life care of the patients with advanced dementia and enhances and reinforces their beliefs and attitudes [35]. Consistent with the results of the current study, Lai X Bin et al. screened delirium patients in need of palliative care in the first stage of which developed four discriminating criteria and one baseline discrimination. Secondly, they identified specific compliance barriers and strategies to promote best practice, and in the third stage they conducted follow-up discriminations that established the best practice for successful delirium screening in the ward [36].

One of the principles of palliative care focuses on patient care so as to relieve pain and other distressing symptoms. Nurses with better self-efficacy in symptom management can better assess patients’ symptoms and effectively contain the negative consequences of those symptoms. Nurse has care capabilities that carefully examine the patient, discern the signs and symptoms of the disease, and deploy creative thinking in specific situations. Patient-centered care education is a priority at all levels of education [37], and almost all studies have shown that education can contribute to improvements in learners’ attitudes, knowledge, skills, and behavior [38–40].

The results of this study revealed the effect of palliative care training on nurses’ “psychosocial support dimension” and adequate education in palliative care escalates the scores of nurses’ psychological support. This dimension of palliative care includes concepts such as communication skills, spirituality, and death. The results of Hamooleh’s et al. [41] study on nurses’ view of spirituality-based palliative care in cancer patients demonstrated that, according to the nurses’ understanding of spiritual concepts, palliative care is important for cancer patients. However, in a qualitative study Jouybari et al. [42] concluded that nurses do not consider diagnostic news delivery, which is one of the dimensions of social psychological support, as part of their responsibility. In another qualitative study, van Leeuwen et al. [43] concluded that from the perspective of nurses, spirituality is one of the critical areas in caring for cancer patients, but they believed this important area was not practically provided to care for such patients. Consistent with the results of this study, Tam et al. examined the effect of a serious illness care-program clinical-education workshop on communication skills (of the components of psychosocial support dimension) of the medical students. Students participated in a 2.5-h workshop the results of which was associated with increased knowledge and self-efficacy [44].

In Iran, since medical education pivots around more symptomatic than holistic care [45], less attention is hence exercised to the basic elements of palliative care such as family support, interdisciplinary teamwork, psychosocial and spiritual distress [46]. Based on the results of the present study, enjoying this educational background can help improve the perceived self-efficacy in the area of psychosocial support.

The pitfalls of the present study include limited time, overwork of the nursing staff, and participants’ moods when responding to the questionnaire. Pre- and post-method design was used in this study, thus lack of any control group was another imitation of the probe. For future investigations, therefore, we recommended using a control group virtually similar in terms of demographic characteristics and all influential factors similar to the test group to be designed and implemented so as to become more confident about the impact of intervention. Another limitation of the study was lack of behavior control and follow-up period thus being recommended to be considered in future.

## Conclusion

The results demonstrated that developing a training program can be effective in improving the perceived self-efficacy of nurses about palliative care. Despite all the limitations mentioned, this study has its own advantages, i.e., noting that a short-term and low-cost training program can be useful in correcting self-efficacy. From this perspective, it can thus be considered by policymakers and health professionals. Relying on scientific findings and designing and implementing management and educational activities in the field of palliative care, nursing managers and educators can provide conditions to improve the self-efficacy of students and nurses and accordingly improve the quality of nursing care in this area.

This is one of the few studies conducted in Iran investigating the perceived self-efficacy of palliative care nurses so it can act as the springboard for the studies to come.

## Data Availability

Sharing the data is not possible due to an agreement with the participants on the confidentiality of the data.
